# Gamification for Internet Gaming Disorder Prevention: Evaluation of a Wise IT-Use (WIT) Program for Hong Kong Primary Students

**DOI:** 10.3389/fpsyg.2019.02468

**Published:** 2019-11-01

**Authors:** Chor-lam Chau, Yvonne Yin-yau Tsui, Cecilia Cheng

**Affiliations:** Department of Psychology, The University of Hong Kong, Pokfulam, Hong Kong

**Keywords:** internet gaming disorder, gaming addiction, problematic internet use, prevention program evaluation, universal strategy, social impact, risky online behavior

## Abstract

Internet gaming disorder and risky online behavior (e.g., cyberbullying, exposure to online violent content) have emerged as serious problems in the digital age. Prevalence rates range from 4% to 40% across the globe, with Asia being one of the hardest-hit regions. To address these pressing problems, our team designed the Wise IT-use (WIT) program, a universal prevention program that (a) enhances students’ awareness of Internet gaming disorder and an array of common risky online behaviors, and (b) equips them with sufficient knowledge to handle such problems. The WIT program design was based on gamification principles and flow theory to enhance users’ motivation and learning experience. A program evaluation study was conducted to assess the social impact of this program in mitigating symptoms of Internet gaming disorder and risky online behavior, and in bolstering emotional well-being. The participants were 248 students aged 7 to 13 from four primary schools in various regions of Hong Kong. They completed validated questionnaires 1 month before and 2 months after participating in the program to evaluate changes in their symptoms of Internet gaming disorder, the frequency with which they displayed risky online behaviors, and their ratings of emotional well-being across the period. The results revealed that both the symptoms of Internet gaming disorder and the proportion of students at risk of the disorder were reduced after the program. The changes observed in students were related to higher levels of positive affect and lower levels of negative affect. Evidence from this study indicates that Internet gaming disorder and risky online behavior are detrimental to the emotional well-being of Hong Kong primary school students. More importantly, the findings demonstrate that our newly developed WIT program can have a social impact in successfully mitigating the symptoms of Internet gaming disorder and enhancing emotional well-being over time. The implications of these findings for the program’s broader impact on society and culture are discussed.

## Introduction

In the digital age, the use of information technology (IT) has brought efficiency and convenience to myriad aspects of daily life (e.g., C. Chaudhry et al., 2006; Chang et al., 2012). For instance, people can easily communicate with friends anywhere in the world at any time using instant messaging applications. Despite these benefits, IT use has also ushered in adverse behaviors and outcomes, such as cyberbullying victimization and password hacking (e.g., Furnell et al., 2015). In addition to risky online behavior, an extreme form of problematic IT use is addiction, of which Internet gaming disorder is the most common type (e.g., Müller et al., 2015; Sigerson et al., 2017a). The issues pertaining to problematic IT use have raised widespread concern among the general public, mass media, and governments worldwide.

### Problematic IT Use: A Prevalent, Emergent Societal Problem

Alongside the global concern over the array of problems related to IT use, these problems have also attracted considerable research interest. Investigations into the prevalence of IT addiction and risky online behavior have found that the prevalence rates of Internet gaming disorder and Internet addiction range from 8% to 17% across nations (e.g., Cheng and Li, 2014; Mak et al., 2014; Müller et al., 2015). Of the various countries included in such reviews, mainland China has the highest prevalence rate of IT addiction.

Higher prevalence rates have been identified for various types of risky online behavior. For example, a recent meta-analysis revealed cyberbullying victimization to have an overall prevalence rate of 20–40% across age groups (Kowalski et al., 2014). The prevalence of meeting strangers online has been reported to range between 4 and 14% among adolescent samples (Valcke et al., 2007, 2011; Dowdell, 2011). Furthermore, in a study of early adolescents in the United States, 66% of male participants and 39% of female participants reported exposure to online pornography (Brown and L’engle, 2009). In another United States sample, 38% of 10- to 15-year-olds were found to have been exposed to violent content online (Ybarra et al., 2008). Finally, 37% of a sample of Chinese adolescents reported having shared their passwords with others (Mak et al., 2014).

### Psychosocial Problems Related to Problematic IT Use

The high incidence rates of IT addiction and risky online behaviors have broader societal implications, prompting researchers to investigate the impacts of these emergent problems on users’ emotional well-being and on society as a whole. With regard to personal problems, a recent meta-analytic review of studies conducted in multiple nations worldwide revealed an inverse association between Internet gaming disorder and emotional well-being (Cheng et al., 2018). Individuals at risk of such disorder tend to experience more episodes of loneliness, insomnia, and concentration problems (Kim et al., 2016), and are five times more likely to exhibit aggression, impulsive behaviors, and suicide attempts than healthy individuals. Moreover, those with Internet gaming disorder tend to manifest more psychiatric symptoms, such as obsession-compulsion and psychosis, than healthy individuals (Kim et al., 2016). Furthermore, people with IGD have reported higher levels of depression, stress, and anxiety, as well as lower life satisfaction (e.g., Bargeron and Hormes, 2017; Sigerson et al., 2017b).

With regard to interpersonal problems, the aforementioned meta-analytic review also indicated that individuals with Internet gaming disorder encounter more difficulties in their interpersonal relations (Cheng et al., 2018). Owing to their overindulgence in online gaming and neglect of significant others, these individuals are susceptible to a range of interpersonal stressors, including social isolation and interpersonal conflict (e.g., Demetrovics et al., 2012; Wartberg et al., 2017). They also tend to frequently lie about their excessive gaming (Lemmens et al., 2015), inevitably leading to arguments and a further deterioration of interpersonal relations. In addition, these individuals tend to be less socially competent than others, and therefore possess insufficient social skills to handle the interpersonal conflicts elicited by their excessive gaming. Taken together, these issues diminish the quality of interpersonal relations for individuals with Internet gaming disorder, further estranging them from their significant others (e.g., Liau et al., 2015; Cheng et al., 2018).

Similar to Internet gaming disorder, risky online behaviors also elicit a range of personal problems. For instance, cyberbullying victimization is positively associated with depression, substance abuse, and suicidal ideation (Hinduja and Patchin, 2010; Gámez-Guadix et al., 2013). More broadly, risky online behaviors, such as sharing online data (e.g., passwords) and hacking, can lead to significant financial losses for individuals and societies. For example, according to a recent multinational survey conducted across 20 countries (Symantec Corporation, 2018), 978 million consumers suffered an aggregate financial loss of US$172 billion from cybercrimes in 2017, with an average loss of US$142 per victim. This survey further revealed a decline in work productivity as a result of the time taken to deal with cybervictimization, with an average of two working days of productivity lost owing to victims’ preoccupation with the incident.

### Current Approaches to Mitigating Problematic IT Use

The deleterious psychosocial and societal impacts brought about by problematic IT use have led a number of mental health professionals to develop and implement dedicated interventions. A multinational systematic review revealed cognitive behavioral therapy to be the most frequently adopted treatment for Internet gaming disorder (King et al., 2017). A more recent meta-analytic review further demonstrated the efficacy of cognitive behavioral therapy for alleviating the symptoms of such disorder and its associated depression and anxiety (Stevens et al., 2019). Another recent study expanded the scope of cognitive behavioral therapy by incorporating the techniques of humanistic therapy, such as empathy and acceptance (Torres-Rodríguez et al., 2019), with the results revealing such an expanded course of therapy to be effective in (a) ameliorating the symptoms of Internet gaming disorder, (b) reducing the amount of time spent gaming, and (c) mitigating comorbid disorders such as depression and anxiety.

In addition to cognitive behavioral therapy, mindfulness meditation and reality therapy have also been found to attenuate the symptoms of Internet gaming disorder and reduce decisional impulsivity, which refers to the tendency to make risky decisions by favoring smaller immediate rewards over larger but postponed rewards (Yao et al., 2017). Instead of treating the problem of Internet gaming disorder holistically, another study focused on treating the specific symptom of cravings through psychoeducation and mindfulness training (Zhang et al., 2016). The findings showed that reducing clients’ cravings for Internet use mitigated their symptoms of Internet gaming disorder.

Compared to Internet gaming disorder, interventions for risky online behavior have received less scholarly attention. Most intervention programs for risky online behavior have specifically targeted the mitigation of cyberbullying, probably due to the high prevalence of this type of risky online behavior (Gaffney et al., 2019). A recent meta-analysis revealed that school-based anti-cyberbullying interventions are generally effective, with notable decreases in cyberbullying perpetration and victimization observed after such strategies were implemented (Gaffney et al., 2019). Another review found that the most effective preventive interventions for cyberbullying are generally those that target not just a single but several social systems, including the peer group, family, school, and community (Ang, 2015).

Psychoeducation is a common intervention program for mitigating cyberbullying. For instance, one psychoeducation program for middle-school students was designed with reference to the theory of planned behavior (Wölfer et al., 2013). This program focused on strengthening students’ social skills (e.g., perspective-taking), improving their online safety awareness (e.g., for online security options), introducing the legal risks and repercussions of cyberbullying, enhancing behavioral control, and providing protective strategies in the event of cyberbullying. This program was found to be effective in mitigating cyberbullying in schools.

Another psychoeducation program was developed and implemented for primary school students (Toshack and Colmar, 2012). In this program, children acquired knowledge about cyberbullying and its deleterious impacts, along with safety and management strategies for Internet use. Enhancing this kind of knowledge was found to reduce children’s susceptibility to cyberbullying. Although these studies have indicated that cyberbullying interventions can reduce this particular type of risky online behavior, the number of studies is limited. Hence, additional research is needed to further inform future school and public policies (Wölfer et al., 2013; Gaffney et al., 2019). Furthermore, no intervention programs have been developed to date for other risky online behavior, such as exposure to online violent content and exposure to online pornography.

All of the aforementioned programs for Internet gaming disorder are indicated interventions, meaning that they target individuals who manifest early signs of, or have already developed symptoms of, a given psychological problem (see e.g., Haggerty and Mrazek, 1994). An indicated intervention is a major type of impact strategy that works to directly tackle the social problems associated with Internet gaming disorder. It differs from a universal intervention, which is applied to the general population rather than to a specific risky or risk-prone group. The latter strategy seeks to deter or delay the occurrence of symptoms, with the ultimate aim of preventing the initial development of Internet gaming disorder.

### Proposal for a New Universal Approach to Prevent Problematic IT Use

Although practitioners generally believe that prevention is better than cure, our literature review indicates that the impact strategy of a universal intervention for preventing Internet gaming disorder has seldom been adopted. Prevention is especially important for this particular social problem because once excessive gaming has become addictive, a substantial amount of time and effort is necessary to overcome the addiction. Empirical evidence shows Internet gaming disorder to be characterized by long-term issues of withdrawal symptoms and relapse (Torres-Rodríguez et al., 2018). Hence, prevention measures should be introduced as early as possible to maximize their effectiveness. Food and Health Bureau (2017) strongly advocates for early intervention for mental health problems, as “the early stage of life presents an important opportunity to promote mental health and prevent mental disorders as up to half of mental disorders in adults surface before the age of 14” (p. 4). Hence, primary school students constitute the ideal intervention point for preventing Internet gaming disorder.

In response to this call for early intervention, our team designed a universal prevention program called Wise IT-use (WIT), which aimed at mitigating the symptoms of Internet gaming disorder and risky online behaviors in the general population of children. For our program, there were no screening of participants prior to program implementation; instead, we adopted a universal impact strategy targeting children without any symptoms of Internet gaming disorder, those at risk of such a disorder, and those who had already developed symptoms. A universal strategy was preferred for children because the incidence of Internet gaming disorder in children is much lower than it is for adolescents and adults (Paulus et al., 2018). Early intervention may be able to reduce the incidence rate or maintain it at a low level into adolescence and adulthood.

In our program design, we adopted the novel training approach of gamification, which has been proven to be effective in enhancing children’s motivation and learning engagement (e.g., Dicheva et al., 2015). More specifically, our psychoeducation program encouraged the engagement of child participants with a series of play-based activities that convey messages about the undesirable consequences of Internet gaming disorder and risky online behavior. These activities also included practical methods for tackling such problems, including online safety measures and engagement in (offline) physical activities during leisure time. In addition, we incorporated flow theory (Nakamura and Csikszentmihályi, 2001) into our program design. Flow theory posits that the learning experience is maximized when learners’ joyfulness is heightened and they are completely absorbed by the learning activity, which also heightens their intrinsic motivation and active engagement in learning (e.g., Gauntlett, 2007). Immersion in a playful environment facilitates learning (e.g., Geithner and Menzel, 2016).

Adopting these theoretical learning approaches, our team constructed a gamified learning system—AIR (Assimilation, Interaction, Reflection)—to make learning playful, fun, and sustainable for child participants. The WIT program was designed on the basis of the three fundamental components of this system. For the assimilation component, learning principles and materials on safe and healthy online behaviors were designed to be “injected” or assimilated into games such that learners acquire knowledge while being immersed in the games. For the interaction component, students were encouraged to interact with and learn from one another through the play-based activities built in to the learning sessions. For the reflection component, the program ensured that students would be guided by facilitators through various activities that called on them to reflect on their previous online experiences. Such engagement and reflection facilitate deep learning and the long-term retention of knowledge (e.g., Geithner and Menzel, 2016).

### Social Impact of Our Proposed Universal Approach to Prevent Problematic IT Use

The high prevalence of Internet gaming disorder makes it a particularly appropriate target for a universal preventive intervention intended to benefit a whole population, rather than just those subgroups or individuals identified to be at risk (Haggerty and Mrazek, 1994). Our proposed universal preventive intervention confers some advantages over indicated interventions. The social impact of our WIT program reflects its (a) low cost per beneficiary, (b) low risks arising from the intervention, (c) applicability to the entire population of the beneficiary group, and (d) effectiveness demonstrated by empirical evidence (Haggerty and Mrazek, 1994).

By adopting a universal preventive approach, the WIT program aimed to meet the needs of Hong Kong society. Food and Health Bureau (2017) urges that “universal prevention needs to be expanded, as nothing works better than reducing the incidence of disorders by preventing new cases from developing” (p. 99). The current demand for mental health services is elevated not only by the substantially increased number of mental health cases, but also by the heightened public awareness of mental health issues. Consequently, more individuals in need are seeking support from mental health professionals, accompanied by higher expectations for timely and effective mental health services (Food and Health Bureau, 2017). All of these factors exert huge pressure on public health service providers in Hong Kong.

To tackle the current surge in demand for mental health services, Food and Health Bureau (2017) acknowledges the impracticality of relying solely on specialist services to cope with the ongoing increase in mental health cases. Such an approach would impose an even heavier workload on specialists, reducing the time that could be dedicated to supporting each individual and thus inevitably decreasing the overall quality of mental health services. As a remedy, the bureau underscores the importance of universal prevention for mental health, and has proposed further developments in this domain (Food and Health Bureau, 2017; Chan, 2019a). Indeed, universal prevention is fundamental to the “Three-Tier Stepped Care Model,” constituting part of the “Tier-1 services,” which “refer to universal prevention, early detection and intervention as well as mental health maintenance that are accessible by children, adolescents and their families in their everyday life through public education, parenting programs, promotional activities in the community or at schools” (Food and Health Bureau, 2017).

In response to the government’s call, we developed the WIT program to achieve social impact by addressing the emergent needs of society and provide timely solutions to pressing societal issues deriving from problematic IT use and Internet gaming disorder. Our psychoeducation program was the first to incorporate both the gamification approach and flow theory into its design. Adopting these innovative approaches in the program design aimed to enhance the engagement of our beneficiaries, students at upper-primary school levels, in the intervention. The beneficiaries who took part in this program learned and practiced skills that fostered self-regulation, online safety, digital citizenship, and interpersonal communication. Through acquiring and applying such skills in their daily life, the goal was for these students to be able to maintain a healthy balance of Internet use and to bolster their mental well-being accordingly.

The effectiveness of our new program was evaluated by a social impact assessment through the use of quantitative methods. Specifically, the social benefits of our new program were measured in three ways: (a) prevention of the development of Internet gaming disorder through a decrease in the risk levels after program participation, (b) reduction of risky online behaviors, and (c) promotion of mental well-being. This study was thus conducted to empirically test these hypothesized effects of the WIT program. Specifically, we predicted that after students had taken part in our program, they would report fewer symptoms of Internet gaming disorder and less risky online behavior. We also expected them to experience better mental health, as indicated by higher levels of positive affect and lower levels of negative affect, social anxiety, and loneliness. If the program evaluation study yielded these desirable findings, we could conclude with confidence that the WIT program could be implemented in a larger number of schools to reach out to more beneficiaries, thus further broadening the program’s impact on society as a whole.

## Materials and Methods

### Participants and Design

Before the study began, we carried out an *a priori* statistical power test to estimate the number of participants required to detect a statistically significant effect. We used the commonly adopted power analysis software G^∗^Power (version 3.1.9.2; Faul et al., 2009). The power analysis revealed that a sample size of 200 would have sufficient power to detect a medium effect size, with a Type I error probability (α) of 0.05 and a statistical power (β) of 95%. As our study included two time points, we anticipated program attrition of around 20%, and thus recruited a larger sample to make sure the final sample size yielded adequate statistical power for hypothesis testing.

We recruited 248 students from four primary schools, each located in one of the major metro regions of Hong Kong (i.e., Hong Kong Island, Kowloon, New Territories East, and New Territories West). Letters containing details of the study were distributed to the parents of all students in these schools. Those parents who allowed their children to take part were asked to sign and return the consent forms before a specified time, normally 4 weeks before the first session of the program started.

Eligible students were those who returned parental consent forms and gave their own consent. More than half of the participants were boys (56%). The average age of the sample was 10.16 years (*SD* = 0.97), with a range of 7–13 years.

To evaluate the potential changes in Internet gaming disorder, risky online behavior, and psychological outcomes over time, we adopted a two-phase longitudinal design that allowed for within-participant comparisons to be made. The data collection sessions took place 1 month before (Time 1) and 2 months after (Time 2) the implementation of the WIT program. As some of the participating students were absent on one or both of the data collection days, 241 (58% boys) and 226 (55% boys) students completed the questionnaires at Time 1 and Time 2, respectively. As the WIT program had a duration of 3 months, the time span between these two data collection sessions was 6 months.

### Measures of Social Impact

#### Symptoms of Internet Gaming Disorder

To assess the symptoms of Internet gaming disorder, we adopted the self-report version of the Korean Internet Addiction Proneness Scale (National Information Society Agency, 2011) because it is the only scale that has been specifically developed and validated for Asian children and adolescents to assess various types of IT addiction (e.g., Kim et al., 2014; Mak et al., 2017). For all 15 of the scale’s items, we replaced the term “Internet use” with “video game playing.” Sample questions included “After I am done playing video games, I want to play video games again” and “I failed to fulfill things I planned to do because of video game playing.” The participants rated each item on a 4-point Likert scale ranging from *strongly disagree* (1) to *strongly agree* (4). The scale demonstrated adequate reliability at both time points in the current study (Cronbach’s α = 0.75 and 0.82 for Time 1 and Time 2, respectively).

The participants were classified into three groups based on the coding scheme used by Mak et al. (2017): the “average gamers” group included participants with a score of 40 or below, the “at-risk gamers” group included those with a score from 41 to 43 inclusive, and the “high-risk gamers” group included those with a score of 44 or above. The coding procedures were conducted automatically using an SPSS macro program written by our team.

#### Risky Online Behavior

As no comprehensive measure of risky online behavior was available, we compiled a questionnaire to assess the six most common types of risky online behavior: cyberbullying perpetration, cyberbullying victimization, exposure to online pornography, exposure to online violent content, meeting strangers online, and sharing online data. Of the 31 items, 28 were extracted from the Risky Online Behavior Inventory developed by Chang et al. (2016), with the remaining three, which measure meeting strangers online, extracted from the survey protocol designed by Livingstone et al. (2011). Sample questions included “I share pictures of myself with a stranger I met online” and “I posted or texted a hurtful comment about an online photo or video of somebody else (for example, made fun of how they look).” For all 31 items, the participants were asked to rate the frequency with which they had engaged in the behavior on a 5-point Likert scale consisting of *never*, *rarely*, *sometimes*, *very often*, and *always*. The scale had a high degree of reliability at both time points (Cronbach’s α = 0.90 and 0.94 for Time 1 and Time 2, respectively).

#### Positive and Negative Affect

The Positive and Negative Affect Schedule for Children – Short Form (Ebesutani et al., 2012) was used to measure both positive and negative affect. This scale was chosen because it is widely regarded as a short and strongly validated tool for assessing emotional well-being among children. It is composed of positive affect and negative affect subscales, both of which contain five items. Sample questions on the positive affect subscale include “happy” and “lively,” sample questions on the negative affect subscale include “sad” and “afraid.” The participants were instructed to describe the extent to which they have experienced each type of emotion during the past month along a 5-point Likert scale ranging from 1 (*very slightly or not at all*) to 5 (*extremely*). The Chinese version for children has been found to have the relevant psychometric properties (Pan et al., 2015). Both subscales were reliable across the two time points (Cronbach’s α = 0.78 and 0.90 for Time 1 and Time 2, respectively).

#### Social Anxiety

Social anxiety was assessed using the revised version of the Social Anxiety Scale for Children (La Greca and Stone, 1993). We selected this scale because it is a popular validated Chinese measure of social anxiety developed for child participants. Sample questions included: “I’m quiet when I’m with a group of kids” and “I worry about what other children say about me.” The measure comprised 10 items, which the participants rated on a 3-point Likert scale reflecting how true the given statement was for them. The reliability and validity of the Chinese version of the scale have been demonstrated previously (Li et al., 2006), and the scale also displayed good reliability at both time points (Cronbach’s α = 0.89 and 0.94 for Time 1 and Time 2, respectively).

#### Loneliness

The Children’s Loneliness Questionnaire (Asher et al., 1984) was adopted to measure the participants’ feelings of loneliness and sense of social inadequacy, because it is the most frequently adopted validated tool for use with children. Sample questions included “I don’t have anyone to play with” and “There’s nobody I can go to when I need help.” This questionnaire contained 16 items, and the participants indicated the extent to which they considered each statement to be true for them on a 5-point Likert scale (from 1 = *not at all true* to 5 = *always true*). The Chinese version has been validated (Yue et al., 2014). In this study, the questionnaire’s reliability was high at both time points (Cronbach’s α = 0.90 and 0.90 for Time 1 and Time 2, respectively).

#### Demographic Information

At the end of the questionnaire, participants reported their sex, age, class, and class number. The class and class number were used to match the data collected at the two time points.

### Universal Prevention Program

The WIT program lasted for 3 months. It consisted of online training and an onsite workshop. During the online training, the student participants were asked to complete a series of online modules on topics such as cybersecurity and digital citizenship. The training program contained three parts. In the first part, the prevalent problems of Internet gaming disorder and risky online behavior were introduced. Participants were then shown the unfavorable consequences of Internet gaming disorder and risky online behavior in the second part. In the final part, participants learned effective ways of combating these problems. All of the online training modules featured multimedia presentations, educational games and inter-class tournaments, animations, pop quizzes, and video discussions.

To consolidate the knowledge acquired in the online training modules and foster real-life knowledge application, a number of interactive learning games and activities were designed and delivered in an offline workshop. We considered it essential for student participants to actively apply what they had acquired in the online training modules in the games and activities conducted in the onsite workshop. Most of the participants applied their knowledge by engaging in gameplay and reported immense enjoyment during the learning process.

### Procedures

The onsite training and data collection sessions were conducted in the schools from which the participants were recruited. Prior to training and data collection, the parents of all participants were provided with complete and detailed information on the study, and the participants and their parents provided consent prior to study commencement. The study protocol received prior approval from the Human Research Ethics Committee of the research team’s university.

In the first data collection session (Time 1), a research assistant oriented the participants, encouraged them to be open about expressing themselves, and provided them with instructions on how to answer the questions using the rating scales. Special guidance was given to participants who had difficulties in reading, understanding the questions, or both. The participants were reassured that their data would be used strictly for research purposes and would not be shown to their family members or to any school personnel. All participants filled in a set of baseline questionnaires. Three to four weeks later, the participants attended a 3-month training program.

Two months after the training program (Time 2), participants were invited to attend a second data collection session during which they filled in the same set of questionnaires. The participants were debriefed at the end of the entire study and thanked for their participation.

### Procedure for Preliminary Analyses and Social Impact Assessment

Before conducting a social impact assessment of the new WIT program, we performed preliminary analyses to explore the potential demographic differences. An independent-samples *t*-test was used to detect possible sex differences, and Pearson product-moment correlation analysis was performed to detect possible age differences. If demographic differences were found, separate analyses would be conducted for each demographic group. In addition, Pearson product-moment correlation analysis was used to indicate the magnitude of the inter-relationships among the study variables.

For the social impact assessment, we adopted the following statistical methods to evaluate the social impact of the WIT program. We first evaluated whether our new program could prevent Internet gaming disorder by detecting the presence of changes in levels of risk before and after the program. Using the scores derived from the measure of Internet gaming disorder, participants were classified into “average gamers,” “at-risk gamers,” or “high-risk gamers” according to a scoring scheme for Asian youngsters (Mak et al., 2017). Their scores at Time 1 and Time 2 were compared to identify any changes in group membership, and the significance of such changes was determined via Pearson’s chi-square tests. These tests were used due to their appropriateness for analyzing group membership, given that it was a quantitative nominal variable (Everitt and Wykes, 1999). In these tests, our program’s social impact would be reflected by a significant increase in the proportion of participants in the “average gamers” group.

We then evaluated whether our new program could reduce the symptoms of Internet gaming disorder and risky online behavior while enhancing mental well-being. To attain this goal, a paired-samples *t*-test was used to assess any significant changes in the scores of the study variables from Time 1 to Time 2. We performed this type of *t*-test because it was the most powerful method for detecting hypothesized within-participant variations between two assessments of quantitative continuous variables measured at different time points (Zimmerman, 1997). The social impact of this program would be indicated by significant reductions in levels of symptoms of Internet gaming disorder, risky online behavior, negative affect, social anxiety, and loneliness, as well as an increase in levels of positive affect over time.

Finally, Pearson product-moment correlation analysis was also used to examine the association between the baseline (Time 1) and follow-up (Time 2) scores for all of the study variables. This analysis was chosen because it was deemed the most stable measure of interdependence among the set of quantitative continuous variables (Gay et al., 2011), offering a direct assessment of the extent to which the variables were associated with each other. The program’s social impact would be shown by significant longitudinal changes in the expected directions. All of these analyses were performed using SPSS Version 23.0.

## Results

### Preliminary Analyses

Independent-samples *t*-tests revealed no significant differences in responses to the study variables according to respondents’ sex and none were significantly correlated with age (*p*s > 0.05). Hence, the major analyses were performed on the pooled sample. Table 1 presents the descriptive statistics of the study variables assessed at both time points.

**TABLE 1:** Descriptive statistics of all the study variables at the two time points.

**Study variable**	**Mean**	***SD***
T1 IGD symptoms	31.69	6.42
T1 risky online behavior	7.82	2.07
T1 positive affect	17.19	4.97
T1 negative affect	8.78	3.71
T1 social anxiety	15.46	4.96
T1 loneliness	37.12	7.22
T2 IGD symptoms	29.31	7.47
T2 risky online behavior	7.50	2.18
T2 positive affect	17.85	5.59
T2 negative affect	9.24	4.55
T2 social anxiety	15.36	5.41
T2 loneliness	38.69	9.51

*IGD = internet gaming disorder; T1 = time 1; T2 = time 2.*

Table 2 summarizes the results of the zero-order Pearson correlations for the pooled sample. As shown in Table 2, symptoms of Internet gaming disorder and risky online behavior were both positively associated with negative affect and social anxiety at Time 1 (*r*s > 0.20, *p*s < 0.002). At Time 2, they were both positively related to negative affect, social anxiety, and loneliness (*r*s > 0.20, *p*s < 0.004), and inversely related to positive affect (*r*s = −0.29 and −0.18, *p*s < 0.009).

**TABLE 2:** Zero-order correlation coefficients among study variables.

**Study variable**	**2**	**3**	**4**	**5**	**6**	**7**	**8**	**9**	**10**	**11**	**12**
1. T1 IGD symptoms	0.39^∗∗^	–0.06	0.29^∗∗^	0.31^∗∗^	0.19^∗∗^	0.53^∗∗^	0.34^∗∗^	−0.15^∗^	0.21^∗∗^	0.25^∗∗^	0.13
2. T1 risky online behavior	–	–0.07	0.25^∗∗^	0.20^∗∗^	0.10	0.28^∗∗^	0.42^∗∗^	–0.19^∗∗^	0.20^∗∗^	0.13	0.06
3. T1 positive affect		–	–0.12	–0.09	0.09	−0.14^∗^	0.04	0.60^∗∗^	–0.10	–0.11	0.07
4. T1 negative affect			–	0.36^∗∗^	0.17^∗∗^	0.31^∗∗^	0.20^∗∗^	–0.24^∗∗^	0.49^∗∗^	0.21^∗∗^	0.17^∗^
5. T1 social anxiety				–	0.27^∗∗^	0.27^∗∗^	0.22^∗∗^	–0.09	0.38^∗∗^	0.56^∗∗^	0.25^∗∗^
6. T1 loneliness					–	0.12	0.26^∗∗^	0.01	0.20^∗∗^	0.13	0.23^∗∗^
7. T2 IGD symptoms						–	0.34^∗∗^	–0.29^∗∗^	0.35^∗∗^	0.32^∗∗^	0.15^∗^
8. T2 risky online behavior							–	–0.18^∗∗^	0.29^∗∗^	0.25^∗∗^	0.20^∗∗^
9. T2 positive affect								–	–0.22^∗∗^	–0.06	0.14^∗^
10. T2 negative affect									–	0.36^∗∗^	0.23^∗∗^
11. T2 social anxiety										–	0.38^∗∗^
12. T2 loneliness											–

*IGD = internet gaming disorder; T1 = time 1; T2 = time 2. ^∗^*p* < 0.05. ^∗∗^*p* < 0.01.*

These findings, which were replicated at both time points, are in line with our hypothesis that symptoms of Internet gaming disorder and risky online behavior would tend to compromise the mental health of primary school students.

### Social Impact Assessment of the WIT Program

**FIGURE 1:**
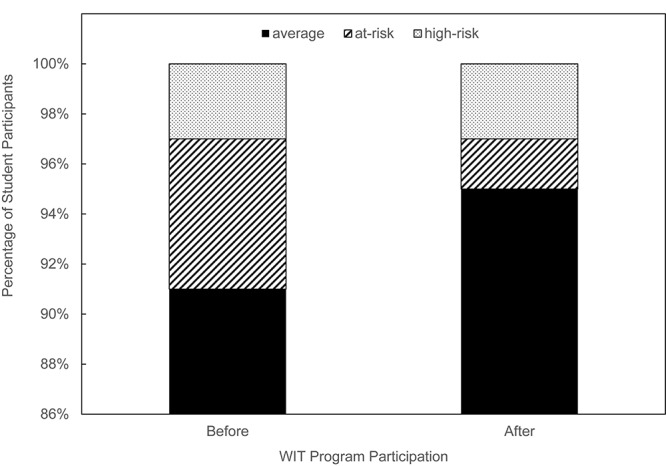
Changes in the proportion of three groups with different severity of Internet gaming disorder symptoms before and after the program participation.

We first assessed the social impact of our new WIT program by examining the changes in the risk of Internet gaming disorder from before (Time 1) to after (Time 2) program participation. We first examined its risk rate before the participants attended the training program (Time 1). According to the aforementioned scoring scheme, 91, 6and 3% of the participants were categorized as “average gamers,” “at-risk gamers,” and “high-risk gamers,” respectively. Then we examined its risk rate after attending the training program (Time 2) to make a comparison of the risk rate between the two time points. A noteworthy result is that 6 months later, the proportion of participants categorized as “average gamers” increased from 91% to 95%, whereas the proportion of those in the “at-risk gamers” group declined from 6% to 2%. However, the proportion of participants classified as “high-risk gamers” remained the same (3%) across the two time points. These changes in group membership are depicted in Figure 1. The chi-square test results revealed these changes to be significant, χ^2^(4) = 42.89, *p* < 0.0001.

A more refined crosstabs analysis further revealed that of the 14 participants classified as being at risk of Internet gaming disorder prior to program participation, 12 were categorized as “average gamers” after participation, with only 2 remaining at risk. Taken together, these results provide some empirical support for the efficacy of our newly developed prevention program in shrinking the group at risk for Internet gaming disorder across the period.

We further assessed the social impact of our program by detecting changes in the trajectory of levels of symptoms and mental well-being from before (Time 1) to after (Time 2) program participation. The paired-samples *t*-test results similarly indicated that the levels of Internet gaming disorder symptoms significantly decreased from Time 1 to Time 2, *t*(203) = 5.16, *p* < 0.0001. However, the reduction in the levels of risky online behavior was only marginally significant across the period, *t*(205) = 1.72, *p* = 0.09.

Finally, to assess the WIT program’s social impact in relieving the symptoms of Internet gaming disorder and boosting mental well-being, the Pearson correlation analyses further showed that having fewer symptoms of Internet gaming disorder at Time 1 predicted lower levels of negative affect and social anxiety at Time 2 (*r*s = 0.21 and 0.25, respectively, *p*s < 0.002) and a higher level of positive affect at Time 2 (*r* = −0.15, *p* = 0.03), and vice versa. Moreover, the exhibition of less risky online behavior at Time 1 was found to predict lower levels of negative affect and higher levels of positive affect at Time 2 (*r*s = 0.20 and −0.19, respectively, *p*s < 0.005), and vice versa.

In summary, these results indicate the social impact of our newly developed WIT program in mitigating symptoms in students at risk of Internet gaming disorder, albeit not for those at high risk. The lack of significant changes in symptom reduction for the high-risk group may be ascribed to the program’s nature, as our program was designed with a preventive focus rather than as an intervention for the treatment of Internet gaming disorder. Our program evaluation study thus provides some empirical evidence for the effectiveness of the WIT program in reducing the risks of Internet gaming disorder for the majority of Chinese primary school students. The results show that the WIT program is conducive to eliciting positive changes in the mitigation of symptoms of Internet gaming disorder, and provide empirical evidence for the social impact of the program in promoting mental and interpersonal well-being.

## Discussion

The results of the social impact assessment demonstrate that our newly developed WIT program is beneficial for the stakeholder group of Chinese primary school students. The social impact of this program received empirical support demonstrating its effectiveness in (a) shrinking the group at risk for Internet gaming disorder, (b) mitigating the symptoms of Internet gaming disorder among primary school students over a 6-month period, and (c) bolstering students’ emotional well-being through increasing levels of positive affect while decreasing levels of negative affect across the same period.

Our findings corroborate the results of previous studies, which have indicated that reduced problematic IT use is related to mental health improvement (e.g., Ceyhan and Ceyhan, 2008; Mei et al., 2016). Taken together, these findings indicate the promising psychological impact of the newly developed program on promoting emotional well-being among primary school students, as the alleviation of problematic IT use mitigates the risks of its comorbid conditions (e.g., depression) and reduces susceptibility to lower levels of self-control and self-esteem, and poorer well-being outcomes (e.g., Ceyhan and Ceyhan, 2008; Mei et al., 2016).

### Evaluation of Program Effectiveness at Three Levels of Prevention

To tackle the societal problems of prevalent problematic IT use and the escalating demands of mental health services, we designed a universal prevention program targeting the general population of primary school students, most of whom had a low to moderate risk of developing Internet gaming disorder. Our prevention program was designed to supplement previous intervention studies, which have focused primarily on tackling mental health issues in individuals who have already developed Internet gaming disorder while neglecting those at risk of the disorder who have manifested some symptoms but not yet developed the condition. As previously noted, the pattern of our findings parallels that of studies examining individuals diagnosed with the disorder. Such empirical consistency indicates that the detrimental mental health and interpersonal consequences of Internet gaming disorder are not limited solely to individuals who have developed the disorder, but are found across a broad spectrum of risk profiles, ranging from very low to very high susceptibility to Internet gaming disorder.

Our study provides evidence indicating that the WIT program is an efficacious universal intervention strategy for both primary and secondary prevention. At the primary level, where the aim is to reduce new incidences of a public health problem (Haggerty and Mrazek, 1994), the program’s effectiveness is substantiated by the post-program increase in the proportion of low-risk students in the current evaluation study. At the secondary level, where the aim is to halt any further increase in established cases of the problem (Haggerty and Mrazek, 1994), the program’s effectiveness is shown by the drop in the proportion of at-risk students. More specifically, those students initially identified as being at risk of developing Internet gaming disorder generally have a low risk after participating in the program.

Despite its effectiveness at both the primary and secondary prevention levels, the WIT program’s psychological benefits are less obvious at the tertiary level, where the aim is to mitigate the symptoms of an existing public health problem (Haggerty and Mrazek, 1994). To elaborate, in the present study the proportion of high-risk students remained the same before and after the program. This unexpected finding is attributable to the preventive nature of our program design. The preventive strategies adopted in the program may be less efficacious for treating Internet gaming disorder because addictive habits require a substantial amount of time and effort to change. Previous systematic reviews have indicated that treating withdrawal symptoms (e.g., restlessness and irritability) and preventing the relapse of Internet gaming disorder require long-term intervention, warranting continuous assessment of the problem and its remission (King and Delfabbro, 2014; Kaptsis et al., 2016). Accordingly, a more comprehensive public health promotion program should incorporate both the WIT prevention program and some existing intervention programs, with the former targeting individuals who may develop Internet gaming disorder and the latter targeting those who have already developed the disorder.

### Social Impact of the WIT Program

The universal approach adopted by our WIT program is conducive to serving a general student population with a relatively wide range of vulnerabilities to Internet gaming disorder, and the program also displays practical utility in exerting a social impact on mental health promotion. As Internet gaming disorder is comorbid with depression, anxiety, attention deficit disorder, and alcohol abuse (Ho et al., 2014), reduced incidences of the disorder are valuable for mitigating these psychosocial and behavioral problems, in turn decreasing the demands on mental health services and the related public expenditures.

#### Impact on Mental Health Service Demand and Related Public Expenditures

Mental health professionals in Hong Kong face immense workloads and work-related pressures, with a ratio of 1 mental health professional to 50 clients with mental illness (Chan, 2019b). This undesirable ratio is largely attributable to the constant escalation in mental health problems over recent years. This escalation is particularly prominent among Hong Kong youths, whose incidence of mental health problems increased by over 50% from 2011 to 2016 (Food and Health Bureau, 2017). Despite the surge in demand for mental health services, the number of psychiatrists and community psychiatric nurses increased by only 3 and 4%, respectively, during the same period.

Long waiting times for treatment are an inevitable consequence of this inflated demand in the face of a shortage in the supply of mental health services. The average waiting time for various child and adolescent psychiatric services increased by 38% over the 2011–2016 period (Research Office of the Legislative Council Secretariat, 2017). Clients and families in need of treatment services often experience considerable distress during the long waiting period. By lowering the incidence of Internet gaming disorder and its related problems, our WIT program can help tackle the long waiting times currently faced by many young Hong Kong clients and their families.

The surging demand for professional services has also generated higher medical costs. In the 2013–2014 financial year, for example, public expenditure on mental health services stood at HK$3.8 billion, increasing to approximately HK$5.1 billion just 5 years later (Chan, 2019a). However, even this markedly elevated expenditure is failing to keep pace with the sharp rise in the demand for mental health services. Providing sufficient services to fully meet that demand would require a substantial increase in public expenditure.

In this light, the primary and secondary prevention strategies adopted by our WIT program are of great practical value in terms of cost-effectiveness. Cost-benefit analyses of mental health services generally indicate that primary and secondary prevention strategies are relatively cost-effective (Harper and Balch, 1975; Barry et al., 2009), as their implementation costs are typically much lower than those of interventions at the tertiary level (Springer and Phillips, 2007). Other findings similarly show that secondary prevention strategies restrict the surge in new cases of psychological problems, which in turn decreases overall expenditure and the need for costlier interventions such as psychotherapy (Durlak and Wells, 1998). These broader benefits of universal programs for the prevention of mental health problems are important, as cost-effectiveness is a crucial criterion for evaluating the feasibility of implementing mental health prevention strategies (e.g., Haggerty and Mrazek, 1994; O’connell et al., 2009).

#### Impact on Various Ecological Systems to Foster Children’s Psychosocial Development

To compile a best practice report for public mental health promotion, it is important to consider the interactions among different ecological systems to meet emergent societal needs. A recent review advocates that “using Bronfenbrenner’s ecological system concepts by clearly considering interactions between and within these systems can result in recommendations that are most useful for guiding public mental health policy and practice” (Eriksson et al., 2018, p. 414). In this light, we propose a system-wide implementation of the WIT program that encourages interactions among various ecological systems (Bronfenbrenner, 2009), at the microsystem (i.e., family and school) and mesosystem (i.e., interconnections between family and school) levels.

Apart from serving students, our WIT program could be expanded to engage teachers and parents. Such an expansion in the scope of the WIT program would incorporate the principles of “scaffolding,” a form of educational practice derived from the sociocultural theory of learning (Gibbons, 2002). Adopting the scaffolding approach, teachers and parents could assist students in the psychoeducation components and activities of our program in an “interactive system of exchange” (Wood et al., 1976, p. 99), enabling students to achieve what is beyond their current ability or what they cannot achieve by themselves (Bakker et al., 2015). Endowing these two stakeholder groups with public health knowledge would invite greater school involvement and augment the ability of parents to strengthen their children’s psychosocial competence.

To attain these goals, teachers could receive training in how to run the WIT program in their schools, thus allowing the program to reach successive cohorts of student beneficiaries. In addition to the formal program, school administrators could also incorporate healthy Internet use into their school curricula; for instance, regular classes could include discussions on such topics as healthy Internet use and ways of managing risky online behaviors. Parents could also be equipped with knowledge to foster the effective monitoring of their children’s Internet use and gaming behavior, and the provision of Internet safety guidance at home.

More broadly, the WIT program may confer further constructive social impacts at the macrosystem level (i.e., culture). As the Internet is intrinsically entwined with culture (e.g., Manovich, 2003; Porter, 2013), the WIT program’s advocacy for reducing problematic IT use and gaming may help to promote positive changes in youth culture. Children and adolescents are “digital natives” who were born into and grew up in a world of IT devices (Teo, 2013), and gaming-related problems are particularly serious for this cohort (Wang et al., 2019). Reducing problematic IT use and gaming would thus free up more time for youngsters to engage in alternative activities in offline contexts with real-life social network members, such as family members and classmates.

The amelioration of problematic Internet behavior may also facilitate the attenuation of “toxic technocultures;” that is, contemporary cultures of anonymous Internet gangs who gather online to harass persons with particular demographic characteristics, such as ethnic and sexual identities (Massanari, 2017). The WIT program endows student participants with opportunities to discuss and reflect on the harmful consequences of toxic cultures for both victims and bullies, and encourages them to stay away from such online groups and forums so as to maintain healthy Internet use, responsible digital citizenship, and good mental health.

## Research Caveats, Future Directions, and Concluding Remarks

Before drawing our conclusions, it is important to raise some caveats about the findings obtained in this pilot study and suggest directions for future research. First, the beneficiaries of our new prevention program were confined to a relatively homogenous group of primary school students residing in Hong Kong. As the resources for this pilot study were limited, the school sample was selected from our team’s list of strategic partners, and participation was entirely voluntary. Once the practical utility of our new program has been established, our team intends to seek extended grants and resources to expand the scope of our community service and research to a much larger pool of schools. To reach out to a greater variety of schools that are more representative of the population, an ideal school sample should be derived from probability sampling based on districts rather than broad regions of Hong Kong (Som, 1995).

With the empirical support provided by the present study, the WIT program can be further implemented with the involvement of additional stakeholder groups. Extension of the program may involve the family and school systems (through, for example, psychoeducation sessions for parents and teachers in addition to students) in Internet gaming disorder prevention to further benefit vulnerable groups of youngsters. Such an extension would be based on the influential role played by parents and teachers in children’s learning through “scaffolding,” a teaching and learning approach to strengthen socio-emotional learning (e.g., monitoring processes) and the psychosocial competence of children (e.g., Gennari et al., 2017; Näykki et al., 2017). Moreover, studies have documented that parental involvement in Internet gaming disorder functions to reduce children’s symptoms of Internet gaming disorder as well as alleviate their gaming-related problems (e.g., Li et al., 2019; Pellerone et al., 2019). Furthermore, extending the WIT program to the school system could help to tackle some of the psychosocial risk factors of Internet gaming disorder, such as poor school connectedness, academic stress, and adverse classmate relations, all of which exacerbate problematic IT use among adolescents (e.g., Li et al., 2013; Wang et al., 2011).

The present social impact assessment yielded encouraging findings for the short- to medium-term benefits conferred by the new WIT program. However, individuals at a high risk of gaming disorder experience the withdrawal symptoms and relapses pertinent to the condition (e.g., Pontes et al., 2014; Kaptsis et al., 2016). A longer assessment period is thus desirable for identifying the possible development of relapse, as the DSM-5 classification of Internet gaming disorder specify that its symptoms must be present for a 12-month period to qualify for a diagnosis (American Psychiatric Association, 2013). Therefore, the length of time covered by the current study may be inadequate for the purpose of tracking the progression and potential re-emergence of this disorder. In the long term, it would be worthwhile to evaluate the therapeutic benefits conferred by our program for a longer period, such as 12 to 36 months, allowing for a more sophisticated investigation of the potential problems of relapse and remission for participants who have shown initial improvement after their program participation (King and Delfabbro, 2014; King et al., 2017). If such a long-term study is not feasible, researchers may use available data to assess the recurrence of symptoms of Internet gaming disorder, with the caveat of the short timeframe (King and Delfabbro, 2014).

To conclude, the WIT program is a universal prevention program developed in response to the Hong Kong SAR government’s call for mental health service provision with reference to contemporary societal needs, specifically the current prevalence of Internet gaming disorder and the escalation in mental health problems among young people. This evaluation study demonstrates that our newly developed program exerts a desirable social impact by mitigating the risks of Internet gaming disorder, along with its concomitant symptoms, and promoting mental wellness among primary school students over time. As a newly proposed program for universal prevention, the evidence-based WIT program has the potential to contribute to the enhancement of dedicated public health services for Internet gaming disorder and to enrich technocultures and educational practices that promote the furtherance of social welfare and the quality of life of young people.

## Data Availability Statement

The datasets generated for this study are available on request to the corresponding author.

## Ethics Statement

The studies involving human participants were reviewed and approved by the Human Research Ethics Committee of the University of Hong Kong. Written informed consent to participate in this study was provided by the participants’ legal guardian/next of kin.

## Author Contributions

C-LC and CC contributed to the conception and design of the study. CC coordinated the data collection process. C-LC and YT conducted the literature review. YT and CC performed the statistical analysis and wrote the sections of the manuscript. C-LC wrote the first draft of the manuscript. All authors contributed to the editing and revision of the manuscript, and approved the submitted version.

## Conflict of Interest

The authors declare that the research was conducted in the absence of any commercial or financial relationships that could be construed as a potential conflict of interest.
